# Hot Deformation Behavior and Microstructural Evolution of PM Ti43Al9V0.3Y with Fine Equiaxed γ and B2 Grain Microstructure

**DOI:** 10.3390/ma13040896

**Published:** 2020-02-17

**Authors:** Dongdong Zhang, Yuyong Chen, Guoqing Zhang, Na Liu, Fantao Kong, Jing Tian, Jianfei Sun

**Affiliations:** 1 School of Materials Science and Engineering, Harbin Institute of Technology, Harbin 150001, China; winterzddvip@163.com (D.Z.); kft@hit.edu.cn (F.K.); tianjing@hit.edu.cn (J.T.); 2 College Vanadium and Titanium, Panzhihua University, Panzhihua 617000, China; 3 Beijing Institute of Aeronautical Materials, Beijing 100095, China; g.zhang@126.com (G.Z.); snailtree@163.com (N.L.)

**Keywords:** PM TiAl alloy, hot deformation, deformation mechanism, microstructure evolution

## Abstract

The hot deformation behavior and microstructure evolution of powder metallurgy (PM) Ti43Al9V0.3Y alloy with fine equiaxed γ and B2 grains were investigated using uniaxial hot compression. Its stress exponent and activation energy were 2.78 and 295.86 kJ/mol, respectively. The efficiency of power dissipation and instability parameters were evaluated, and processing maps at 50% and 80% strains were developed. It is demonstrated that the microstructure evolution was dependent on the temperature, strain, and strain rate. Both temperature and strain increases led to a decrease in the γ phase. Moreover, dynamic recrystallization (DRX) and grain boundary slip both played important roles in deformation. Reasonable parameters for secondary hot working included temperatures above 1100 °C but below 1200 °C with a strain rate of less than 1 s^−1^ at 80% strain. Suitable hot working parameters at 50% strain were 1150–1200 °C/≤1 s^−1^ and 1000–1200 °C/≤0.05 s^−1^.

## 1. Introduction

TiAl alloys are considered as a novel type of lightweight material. They have extensive application prospects in the aerospace industry [1,2,3,4,5]. However, due to low ductility and fracture toughness at room temperature, the application of TiAl alloys is limited [6,7]. Great efforts contributed to improving the mechanical properties of TiAl alloys using many methods, such as alloying, as well as thermomechanical and heat treatment [8,9,10,11].

Conventional TiAl alloys mainly consist of an α_2_ and γ phase, which makes them deformable only in a canned or near isothermal environment [12,13]. It was reported that the β phase could improve the hot workability [14,15,16], because the β phase with a disordered body-centered cubic (bcc) crystal structure is favorable for deformation, owing to its 12 independent slip systems, and the β phase is a soft phase at high temperatures. Many researchers demonstrated that V is a strong β stabilizer, as V could promote the formation of β, and good hot workability could be achieved [17,18,19]. In TiAl alloys with less than 45 at.% Al, the solidification pathway occurs through the β phase field, which might consist of the α_2_ + γ, α + γ, α, α + β, and β phases [20]. All TiAl alloys are basically composed of three phases: γ phase, α_2_ phase, and B2 phase. Furthermore, V and Nb are both β stabilizers. They can effectively promote the generation of the β phase. They can all be converted into a certain Cr equivalent. Therefore, no matter whether V or Nb is added, the type of TiAl phase will not change, and the phase transition relationship will not change. In this study, TiAl alloys with the Nb element can be used as a reference object [21].

β is a softening phase in TiAl alloy, which is beneficial for the deformation of the alloy at high temperature, and the V element is a β stabilizer, which can promote the formation of the β phase. Therefore, a high-V TiAl alloy was selected [22]. In addition, Y as a rare earth element can effectively refine the microstructure of the TiAl alloy [22]. Finally, Ti43Al9V0.3Y was identified as the alloy composition for the research. The β phase is very beneficial for the high-temperature deformation ability of TiAl alloys. However, TiAl alloys are still difficult to deform. Thus, it is very necessary and meaningful to investigate the deformation behavior. A constitutive model could accurately reflect the stress–strain of the material during hot deformation [23,24,25]. It plays an important role in predicting and controlling the deformation. After many years of development, constitutive models were adapted to the analysis of various metals, alloys, and intermetallics. They can basically be divided into three types [26]: phenomenological constitutive models, physical-based constitutive models, and artificial neural networks [27,28,29]. Phenomenological models are commonly used in actual material deformation studies because they rely on actual experimental parameters and they have appropriate accuracy. The combination of the Arrhenius formula and the phenomenological model, first proposed by Sellars et al. [30], was successfully used to study the deformation behavior and high-temperature flow stress of various materials, including TiAl [31,32], Ti alloy [33,34], Mg alloy [35,36], and Al alloy [37,38]. Therefore, the combination of the Arrhenius formula and the phenomenological model was used in this study.

The definition and method of the processing map were originally proposed by Raj [39], and it is affected by the microstructure development and reconstruction mechanism. In the present study, the approach of a processing map based on the principles of the dynamic material model (DMM), proposed by Prasad [40], is used to understand the hot deformation mechanism of powder metallurgy (PM) Ti43Al9V0.3Y alloy. The processing map usually consists of an instability map and a power dissipation map. The processing map can effectively optimize the hot processing parameters, while it can also guide the actual deformation.

Additionally, the hot deformation activation energy Q is an important indicator usually used to evaluate the deformation ability of materials. In previous studies, Q was usually taken as a constant, independent of the hot deformation conditions. In fact, Q is greatly affected by the hot deformation parameters. Thus, a clear understanding of the Q of TiAl alloys is actually crucial to reveal the deformation mechanism.

The plastic deformation behavior of PM Ti43Al9V0.3Y was studied based on the flow stress–strain curves tested by hot compression under different temperatures and strain rates. In order to obtain an optimal hot working window, the processing map and Q were developed, and the microstructure evolution was observed and analyzed.

## 2. Materials and Experimental Procedure

**Figure 1:**
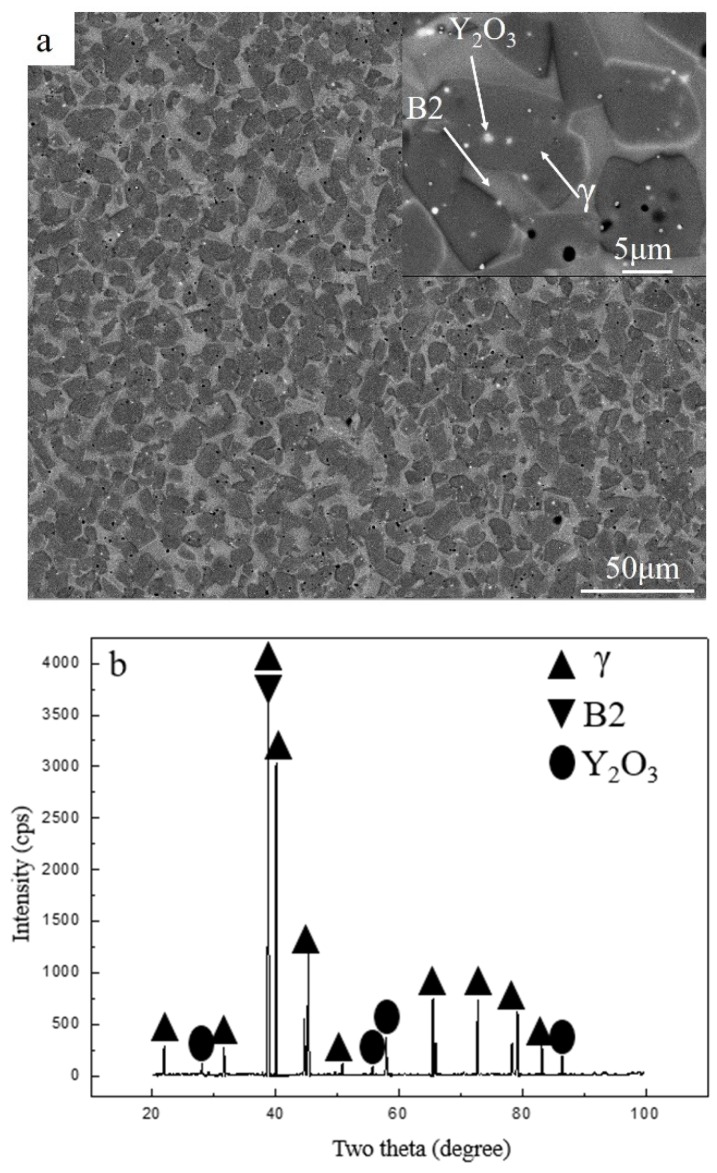
The microstructure (**a**) and X-ray diffraction (XRD) pattern (**b**) of the initial material.

TiAl was prepared by powder metallurgy, under hot isostatic pressing (HIP). Powder with a particle diameter of ≤80 µm was chosen. The N and O contents of TiAl were 140 ppm and 760 ppm. HIP was performed in an argon atmosphere at a temperature of 1200 °C, with a pressure of 140 MPa, for 5 h. The nominal composition of HIP TiAl was Ti43Al9V0.3Y, which was tested by X-ray fluorescence (XRF, Panalytical Analytical Instruments, Almelo, Netherlands); the results are shown in Table 1. X-ray diffraction (XRD, Panalytical Analytical Instruments, Almelo, Netherlands) and scanning electron microscopy (SEM, Field Electron and Ion Company, Hillsboro, OR, USA) were used to characterize the microstructure and phase composition, with the results shown in Figure 1. The microstructure and the phase of hot compression samples were characterized by scanning electron microscopy (SEM), electron backscatter diffraction (EBSD, TSL OIM Analysis 6, Hillsboro, OR, USA), and transmission electron microscopy (TEM, Field Electron and Ion Company, Hillsboro, OR, USA). The step size applied for scanning in the EBSD test was 0.5 μm. The phase analysis of the initial material was mainly based on the backscattered mode of the scanning electron microscope and related literature [41]. The Ti43Al9V0.3Y alloy is mainly composed of the B2 phase (International Centre for Diffraction Data file numbers: 44-1288) and γ phase (International Centre for Diffraction Data file numbers: 05-0678). The microstructure of the initial material consists of γ and B2, and many white submicron Y_2_O_3_ particles (International Centre for Diffraction Data file numbers: 43-1036) are distributed in microstructure, especially in γ grains and at grain boundaries. The compressive samples are cylindrical of 8 mm in diameter and 12 mm in length. The hot compression tests were performed on a Gleeble-1500D thermomechanical simulator in a wide range of temperatures and strain rates, as shown in Table 2. The deformation behavior and microstructure evolution were studied, so as to provide a basis for practical forging and rolling deformation. Therefore, the hot compression parameters were roughly in the appropriate hot working window. The TiAl alloy in this study contained a high β phase composition. The β phase will transform into ordered B2. Kumpfert found that the order/disorder transition temperature of the B2/β phase is approximately 1100 °C [42]. Other researchers believe that the temperature is 1175–1200 °C [43]. Therefore, it can be speculated that the order/disorder transition temperature of B2/β should be between 1150 and 1200 °C. When the temperature is too high, the material enters the single-phase region and the grain grows rapidly. When the temperature is too low, the brittle B2 remains in the microstructure. Both conditions can hinder material deformation or even cause cracking. TiAl alloys are suitable for hot deformation in the two-phase region. In this study, the processing of the two-phase region was roughly carried out between 1000 and 1200 °C. At the same time, TiAl alloys are sensitive to strain rate, and the strain rate cannot be too fast. To systematically study the deformation behavior from low to high strain rate, a strain rate of 0.001–1.000 s^−1^ was selected. In general, the deformation of TiAl alloys reaches 30%–90%. Therefore, two representative deformations were selected: 50% and 80%. In order to reduce the friction effect, graphite sheets were used for lubrication. However, the friction would still affect the stress–strain curve. Considering this factor, the stress–strain curves were frictionally corrected using the following equations [44]:

(1) $$\sigma =\frac{{\mathrm{Pc}}^{2}}{2\left(\exp\left(c\right)-2c-1\right)},$$

(2) $$c=\frac{2\mu r}{h},$$

where σ is the stress after friction correction, P is the raw stress data output by the machine, r and h are the instantaneous radius and height of specimen, and µ is the friction coefficient, which is determined according to the amount of barreling for each specimen [45]. Specifically, the barreling factor is defined by the following equations [45]:

(3) $$b=4\frac{\Delta R}{R}\frac{h}{\Delta h},$$

and

(4) $$\mu =\frac{\frac{R}{h}b}{\frac{4}{\sqrt{3}}-\frac{2b}{3\sqrt{3}}},$$

where b is the barreling factor, ΔR is the difference between the maximum and minimum radius, R is the radius after the sample is deformed under the assumption of no friction (determined by assuming a constant specimen volume), h is the final height, and Δh is the height difference between the initial and deformed specimen. The corrected true stress–strain curve can accurately reflect the dynamic response of the material during thermal deformation.

**Figure 2:**
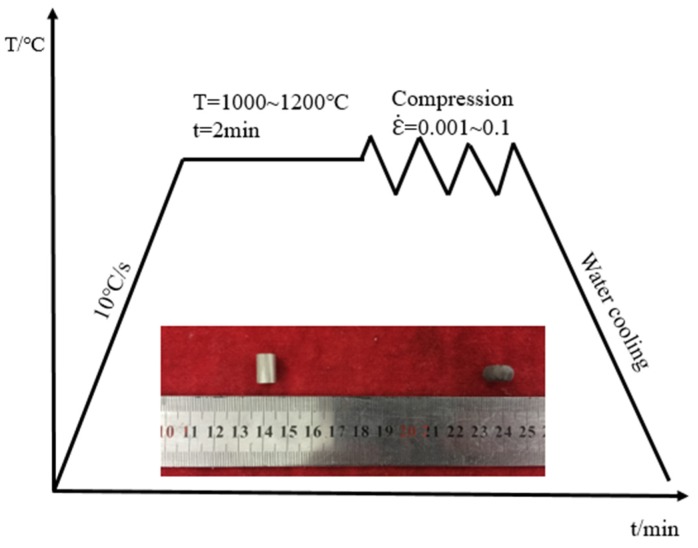
Schematic illustration of the compressive deformation processes.

A thermocouple spot welded at the center of the sample surface was used to monitor the temperature. A schematic illustration of the compressive deformation processes is shown in Figure 2. Before hot compression, all samples were heated up to the test temperature at 10 °C /s, which was held for 2 min to homogenize the sample’s temperature. During hot compression, the thermomechanical simulator continuously collected and organized the flow stress under different thermal deformation parameters through a computer equipped with an automatic data acquisition system. At the end of the compression, the deformed samples were immediately water-quenched to preserve the deformed microstructure. The hot-compressed specimen was cut along the compression axis. A microstructure investigation was conducted on a scanning electron microscope (SEM) equipped with electron backscattered diffraction (EBSD) and a transmission electron microscope (TEM).

The samples for SEM, EBSD, and TEM were prepared by successive mechanical polishing with SiC papers up to 2000 mesh. The SEM and EBSD samples were electrolytically polished before observation. The composition of the polishing solution was 60% methanol, 30% *n*-butanol, and 10% perchloric acid. The polishing parameters were −25 °C and 30 V. The TEM samples were obtained by ion thinning.

## 3. Results and Discussion

### 3.1. Flow Behavior

**Figure 3:**
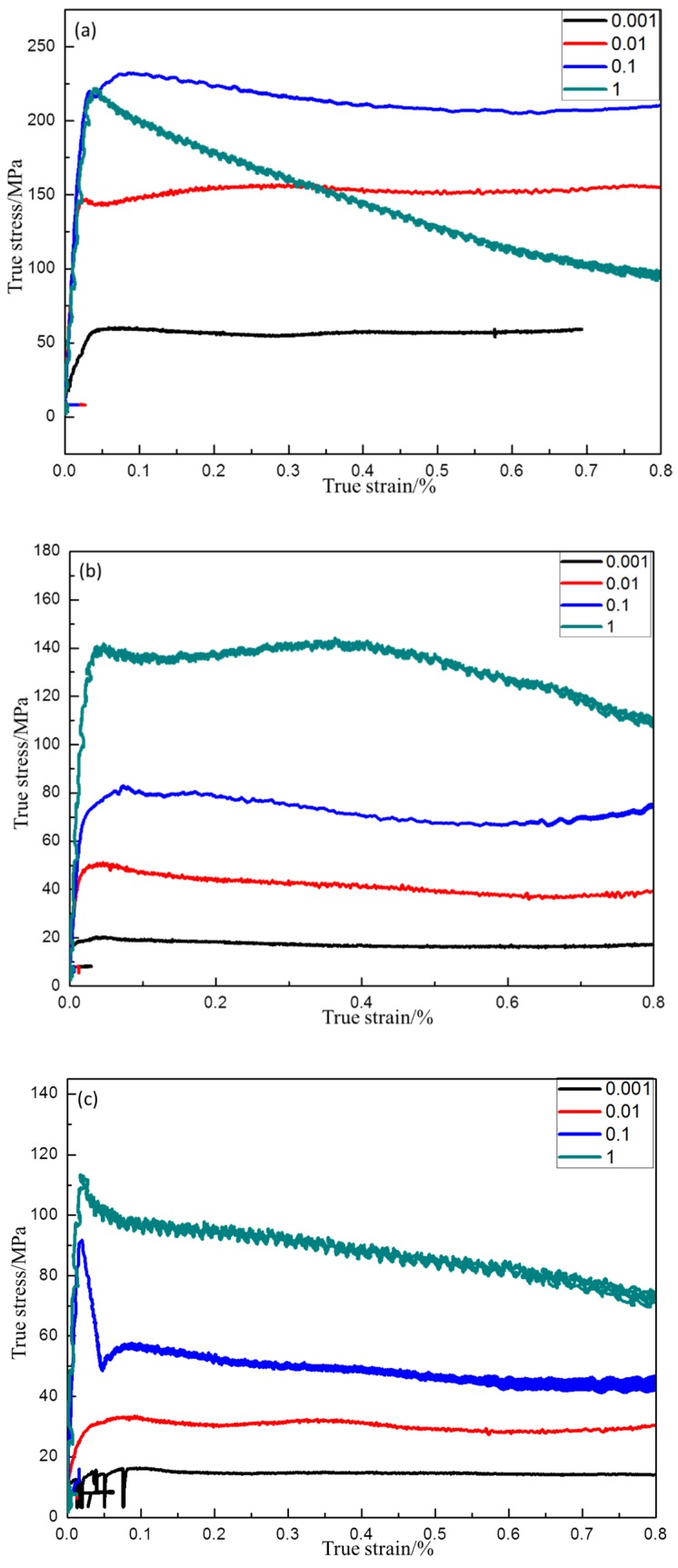
Flow stress curves of powder metallurgy (PM) Ti43Al9V0.3Y alloy deformed in compression at 80% strain/0.001 s^−1^–1 s^−1^: (**a**) 1000 °C (**b**) 1100 °C, (**c**) 1200 °C.

After hot compression, the flow stress–strain curves were obtained. In Figure 3a, the peak stress reached 231 MPa when the specimen was deformed at 1000 °C/0.1 s^−1^, whereas the peak stress was only 59 MPa when the strain rate was 0.001 s^−1^. The former value is approximately four-fold larger than the latter value. This indicates that flow stress decreased drastically with decreasing strain rate. In general, almost all stress–strain curves exhibited the following characteristics: at the beginning, stress increased very quickly. This could be attributed to the fluctuation of the dislocation density. The deformation behavior was the result of competition between dynamic softening and work hardening at elevated temperature. In the early stage of deformation, dislocation density increased rapidly. When work hardening exceeded the dynamic softening, the stress–strain curve rose sharply. Subsequently, as the strain increased, the accumulated energy stimulated the generation of dynamic recrystallization (DRX). DRX eliminated the accumulation of dislocations and alleviated the work hardening effect. Then, the stress–strain curve slowly decreased. Finally, work hardening and dynamic softening reached a state of dynamic equilibrium, and a steady stress state was obtained. According to the literature [46], after the stress reaches its peak, under the action of dynamic recovery (DRV), the dynamic softening speed exceeds the dynamic hardening speed. The stress level gradually decreases, and finally the dynamic softening and dynamic hardening reach an equilibrium state. However, low-stacking-fault-energy materials sometimes undergo discontinuous dynamic recrystallization (DDRX) when conditions are suitable. In Figure 3, the material accumulated a large amount of energy at a rapid strain rate of 1 s^−1^, reaching the energy threshold required by DDRX. Therefore, under the combined action of DRV and DDRX, the softening speed was always greater than the hardening speed, leading to a decrease after the value of 0.8 for the true strain in Figure 3a. In Figure 3b, stress decreased after reaching the steady state. This was due to the discontinuous yielding mechanism [47]. There are two theories to explain this phenomenon [48]. The first is the static theory, which involves the locking and unlocking of dislocations. In the beginning, the dislocations are locked by the solute atoms. When the stress is high enough, the dislocations start suddenly. This causes a reduction in stress. The second is the dynamic theory. This is related to the sudden rise of new mobile dislocations. These new mobile dislocations originate from the grain boundaries [49].

At the same strain rate, the flow stress decreased significantly with increasing temperature (Figure 3a–c), showing negative temperature sensitivity. This might be because, at a higher temperature, the increased external energy enables the material to reach the threshold for DRX at a lower dislocation density. Figure 3 demonstrates that flow stress is dependent on temperature, strain, and strain rate. For all stress–strain curves, when the strain was relatively small, the flow stress increased sharply. After peak stress, the flow stress decreased slowly and eventually remained constant. The characteristics of the flow stress–strain curves were related to the dynamic softening effect.

**Figure 4:**
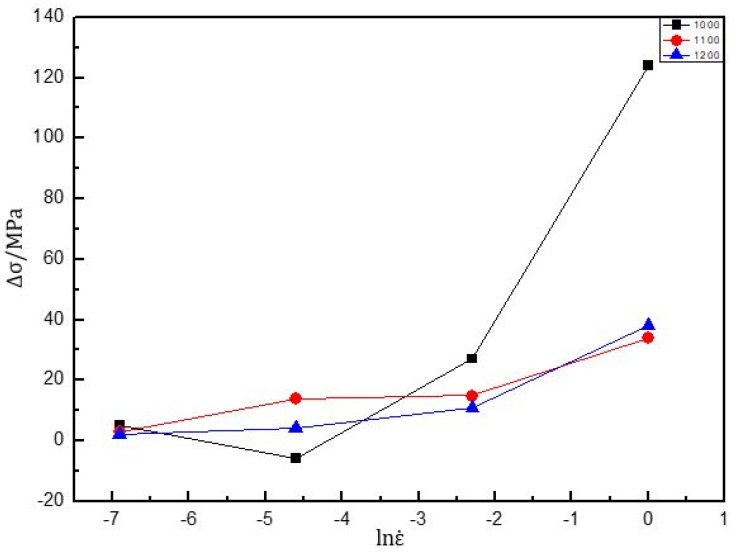
The softening of flow stress under different deformation conditions for the PM Ti43Al9V0.3Y alloy.

In order to describe the dynamic softening extent during deformation, a method to evaluate the temperature and strain rate effect on the softening extent is introduced (Δσ = σ_p_ − σ_s_, σ_p_ represents the peak flow stress and σ_s_ represents the steady-state flow stress when the stain is 80%). Figure 4 shows that the softening extent varied with strain rate and temperature, indicating that the material was sensitive to strain rate and temperature. At the same temperature, the softening extent increased as the strain rate increased. When the strain rate was constant, the softening extent decreased as the temperature increased. In addition, a maximum flow softening of about 124 MPa could be observed at 1000 °C/1 s^−1^. Moreover, a minimal flow softening of 2 MPa was obtained at 1200 °C/0.001 s^−1^, indicating that σ_p_ and σ_s_ stresses were almost the same at high temperature and low strain rate. The softening was the result of competition between dynamic softening and work hardening. At high strain rates and relatively low temperatures, the dislocation network was rapidly accumulated at the initial stage. Due to the relatively low temperature, the material needed to store more strain energy to reach the threshold of dynamic softening. Thus, the flow stress increased to a higher level. When dynamic softening exceeded the work hardening, the flow stress decreased gradually, which was attributed to DRV (dynamic recovery) or DRX (dynamic recrystallization). At low strain rates and high temperatures, the dislocations were easier to initiate, and the material was more likely to reach the dynamic softening threshold.

### 3.2. Activation Energy

The activation energy obtained by the kinetic analysis during hot deformation is an important indicator to judge the difficulty of processing. It could guide the optimization of the deformation. Additional information on the microstructure evolution and the flow stress curve is provided. The Arrhenius-type constitutive model reveals an intrinsic correlation between the true stress and deformation parameters including strain, strain rate, and temperature. This equation is usually expressed as follows:

(5) $$\dot{\varepsilon }=A_{1}\sigma^{n_{1}}\exp\left(-\frac{Q}{\mathrm{RT}}\right)\left(\alpha \sigma <1.2\right),$$

(6) $$\dot{\varepsilon }=A_{2}\exp\left(\beta \sigma \right)\exp\left(-\frac{Q}{\mathrm{RT}}\right)\left(\alpha \sigma >1.2\right),$$

(7) $$\dot{\varepsilon }=A{\left[\sinh\left(\alpha \sigma \right)\right]}^{n}\exp\left(-\frac{Q}{\mathrm{RT}}\right)\left(\mathrm{all}\;\alpha \sigma \right),$$

where $\dot{\varepsilon }$ is the strain rate, T is the absolute temperature, R is the gas constant (8.314 J·mol^−1^·K^−1^), and Q is the activation energy. A_1_, A_2_, A, α, β, n_1_, and n are temperature-independent material constants. By taking the natural logarithm of the both sides of the Equations (5)–(7), one can obtain

(8) $$\ln\;\dot{\varepsilon }=\ln A_{1}+n_{1}\ln\sigma -\frac{Q}{\mathrm{RT}},$$

(9) $$\ln\;\dot{\varepsilon }=\ln A_{2}+\beta \sigma -\frac{Q}{\mathrm{RT}},$$

(10) $$\ln\;\dot{\varepsilon }=\ln A+n\ln\sin h\left(\alpha \sigma \right)-\frac{Q}{\mathrm{RT}}.$$

By taking the partial differential, the material constants n_1_, β, n, and Q can be calculated as follows:

(11) $$n_{1}={\left(\frac{\partial \ln\;\dot{\varepsilon }}{\partial \ln\sigma }\right)}_{T},$$

(12) $$\beta ={\left(\frac{\partial \ln\;\dot{\varepsilon }}{\partial \sigma }\right)}_{T},$$

(13) $$n={\left(\frac{\partial \ln\;\dot{\varepsilon }}{\partial \ln\left(\sin h\left(\alpha \sigma \right)\right)}\right)}_{T},$$

(14) $$\alpha =\beta /n_{1}$$

(15) $$Q=\mathrm{Rn}{\frac{\partial \ln\left(\sinh\left({\alpha \sigma }_{p}\right)\right)}{\partial \left(1/T\right)}|}_{\dot{\varepsilon }},$$

where n is the work hardening exponent, which represents the material’s uniform deformation ability. Hence, Q and n could be calculated to be 295.86 kJ/mol and 2.78, which are lower than the values obtained for self-diffusion of Al in γ-TiAl (360 kJ/mol) but higher than those for self-diffusion of Ti in γ-TiAl (250 kJ/mol). Furthermore, these values are also lower than the Q (342.27 kJ/mol) of as-forged Ti43Al9VY alloy, PM Ti46Al4Nb2Cr2Mn alloy, and as-cast Ti46Al2Cr4Nb0.2Y alloy. The Q is the threshold of the plastic deformation. A lower Q facilitates deformation, enabling dislocation movement and DRX, while exhibiting more desirable hot workability. Some studies considered that a lack of solid solution-strengthening alloy elements, like Nb, is the cause of the low Q. On the other hand, grain boundaries are a type of weak area, which are more prone to deformation at a high temperature. In this study, the mean grain size of starting materials approached 10 μm (Figure 1a), which is very fine. The microstructure contained a large number of grain boundaries, which were conducive to the high-temperature deformation.

### 3.3. Hot Processing Map

**Figure 5:**
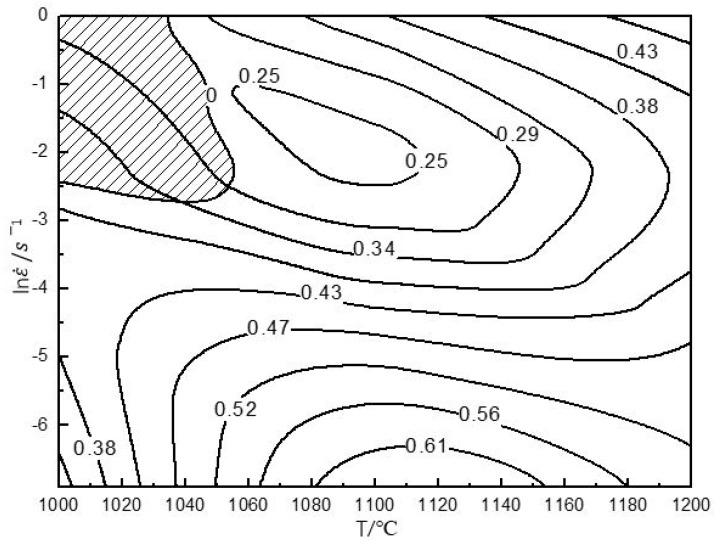
The processing maps of the PM Ti43Al9V0.3Y alloy at 50% strain.

**Figure 6:**
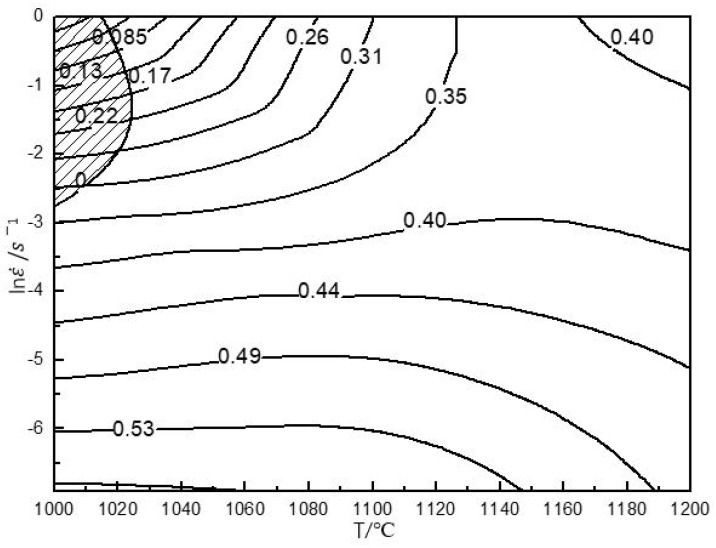
The processing maps of the PM Ti43Al9V0.3Y alloy at 80% strain.

The hot processing maps with 50% and 80% strains are shown in Figure 5 and Figure 6, respectively. The characteristics of the two hot processing maps were nearly the same. They exhibited an unstable domain in the following temperature and strain rate ranges: (1) 1000 °C–1040 °C/0.1 s^−1^–1 s^−1^, and (2) 1000 °C–1030 °C/0.1 s^−1^–1 s^−1^. Both unstable domains were small, indicating that the material had a wide hot working window. The deformation in the unstable domain would cause cracking. In Figure 5, under the 50% strain, ≤1040 °C/0.1 s^−1^–1 s^−1^ represented the unstable domain. Therefore, it was not suitable as a processing domain. Thus, strain rates higher than 0.1 s^−1^ and temperatures lower than 1040 °C were not desirable for hot working. However, despite the above unstable domain, the material exhibited good deformation ability owing to some dynamic softening mechanisms like DRX, as shown in Figure 3 and Figure 4. Rao [31] found that niobium-rich intermetallic PM Ti46Al4Nb2Cr2Mn resisted deformation, which would hinder the deformation of other phases. However, the material contained a high B2 phase composition (Figure 1a), and the B2 phase transforms to the β phase at elevated temperature, where the order/disorder transition temperature of the B2/β phase is approximately 1100 °C. As a soft phase, the β phase could promote high-temperature deformation.

Similar results in terms of processing maps were reported for other TiAl alloys [50,51,52,53]. The hot processing map of Ti45Al8Nb2Cr2Mn0.5Y [54] indicated that the deformation mechanism for a higher efficiency of power dissipation (η ≥ 0.55) was DRX, corresponding to a temperature range of 1000–1200 °C and a strain rate range of 0.001 s^−1^–0.01 s^−1^ at 50% strain. The DRX preferably occurs with high η, corresponding to a high temperature and low strain rate.

In addition to these unstable domains, the map exhibited two high-peak-efficiency domains at 50% and 80% strain. At 50% strain, the high-peak-efficiency domain occurred at 1050 °C–1200 °C/0.001 s^−1^–0.02 s^−1^ with a peak efficiency of approximately 0.43–0.61 (see Figure 5). At 80% strain, the high-peak-efficiency domain occurred at 1000 °C–1200 °C/0.001 s^−1^–0.03 s^−1^ with a peak efficiency of approximately 0.40–0.57 (see Figure 6). Generally speaking, the η associated with DRX is approximately 0.30–0.55 [55]. The η represents the amount of energy used for microstructure evolution. A larger η denotes that more energy is used for microstructure evolution. At 80% strain, in Figure 5, when the temperature exceeded 1100 °C, the η exceeded 0.3. This means that the material was more prone to microstructure evolution. Microstructure evolution under high-temperature deformation often involves DRV or DRX. They would both help deformation. Because the maximum strain rate was 1 s^−1^ in this study, reasonable parameters for secondary hot working were above 1100 °C with a strain rate of less than 1 s^−1^ at 80% strain. Similarly, in Figure 6, at 50% strain, the domain where η exceeded 0.3 was found. Moreover, when the temperature was between 1150 and 1200 °C, the brittle B2 was transformed into β, which was favorable for deformation. Therefore, suitable processing domains at 50% strain were 1150 °C–1200 °C/≤1 s^−1^ and 1000 °C–1200 °C/≤0.05 s^−1^.

### 3.4. Deformed Microstructures

**Figure 7:**
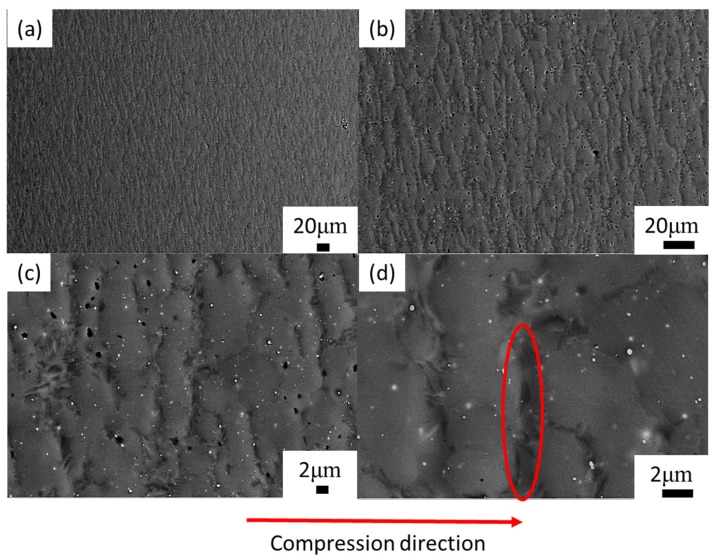
Microstructures of Ti43Al9V0.3Y specimen deformed in compression at 80% strain 1200 °C/0.1 s^−1^ in backscattered mode: (**a**) 200×; (**b**) 500×; (**c**) 2000×; (**d**) 5000×.

**Figure 8:**
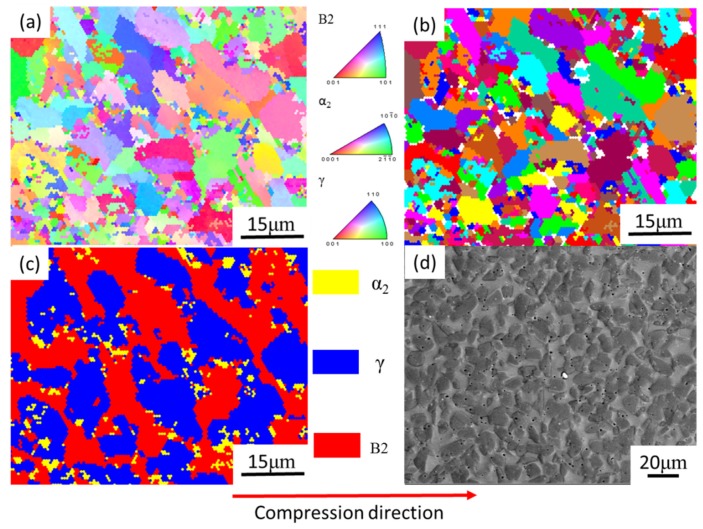
Electron backscatter diffusion (EBSD) phase maps of Ti43Al9V0.3Y specimen deformed in compression at 50% strain/1100 °C/0.1 s^−1^: (**a**) map of inverse pole figure; (**b**) grain map; (**c**) map of phase; (**d**) SEM in backscattered mode.

**Figure 9:**
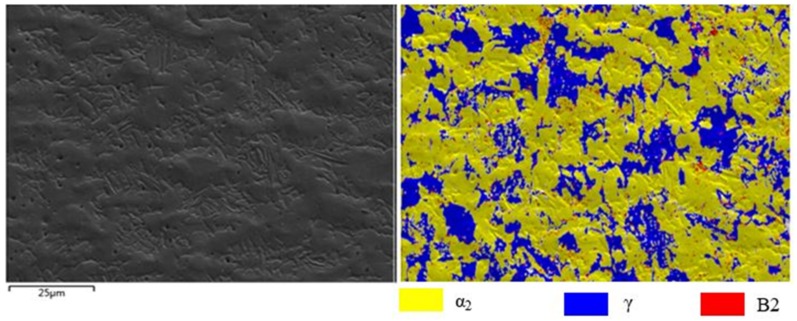
EBSD phase maps of Ti43Al9V0.3Y specimen deformed in compression at 50% strain/1200 °C/0.1 s^−1^.

Microstructure evolution mainly includes DRV (dynamic recovery), DRX (dynamic recrystallization), and phase transformation at elevated temperature. The microstructure is presented in Figure 7, corresponding to 80% strain/0.1 s^−1^/1200 °C. The red arrow is the compression axis, which also applies to subsequent figures. In the high-temperature domain, the grain deformation was obvious (Figure 7a–c). The microstructure consisted of long grains (worm-like) and a transitional grain boundary (Figure 7d), and the grain long axis was perpendicular to the compression axis. The grain length was 34 ± 3.2 μm and the width was approximately 6 ± 0.9 μm. The length-to-diameter ratio was approximately 5:1. The transitional grain boundary’s width was approximately 1 μm (as shown in Figure 7d). When as-forged Ti44Al8Nb (W, B, Y) [56] deformed at 1200 °C/0.1–0.5 s^−1^, the volume fraction of the recrystallization phase increased as the strain rate decreased. In Figure 8, when the material deformed at 1100 °C/0.1 s^−1^, the deformed microstructure was mainly composed of the B2 phase (45%) and γ phase (48%), while it contained only a small amount of the α_2_ phase (7%) (Figure 8c). Using Image Pro Plus (IPP) software to calculate the content of each phase in Figure 1, the content of the B phase was 26%. Compared to the initial material, the B2 phase content increased from 26% to 45%. This implies that, when TiAl was deformed at 1100 °C, a large amount of the γ phase was transformed into the β phase. Zhou reported that β and γ follow a certain orientation relationship during deformation, i.e., {111}γ//{101}β, <101>γ//<111>β [56]. γ transformed into the α phase at around 1125 °C [57], and, as the temperature continued to rise to 1150–1200 °C, and the α phase transformed into the β phase [58]. As reported by Zhou [56], γ and β follow a certain orientation relationship. γ→β transformation is achieved through γ→α→β. When the temperature was 1100 °C, solid-state phase transformation occurred, i.e., γ→β, where part of the γ phase was changed to the β phase. β is a disordered phase of B2 at elevated temperature. As a soft phase, β could promote deformation, and it served as the main deformation phase of TiAl alloys. After the deformation was completed, as the temperature decreased, a solid phase transition occurred again: β→γ + α. Finally, a deformed microstructure consisting of the β phase and γ phase, containing a small amount of the α phase, was obtained. In Figure 8a, the inverse pole figure map shows the grain orientation in the microstructure. In Figure 8b, the grain diagram shows that the microstructure was mainly composed of small equiaxed grains, and there were many smaller grains between and within the grains. These smaller grains were recrystallized grains. When TiAl alloy is deformed, recrystallization can soften the material, which is beneficial to deformation. The IPP software was used to calculate the percentage of small holes before and after the deformation of TiAl. Before TiAl deformation, small holes accounted for 2.1% ± 0.1% of the surface. After deformation, small holes accounted for 1.9% ± 0.2% of the surface. The percentage of holes hardly changed. The reason may be that, during electropolishing, the electrode potential of these precipitates differed from that of the surrounding matrix. The joint between the particle and the matrix was rapidly corroded. Consequently, a part of the particle fell off the matrix and formed small holes in the microstructure. Figure 9 shows the typical microstructure and phase distribution, corresponding to 50% strain/0.1 s^−1^/1200 °C. The microstructure mainly consisted of γ and α_2_, with a little B2. When the material deformed at 1200 °C/0.1 s^−1^, the elevated temperature caused softening inside the grains, and the grains and grain boundaries both deformed [22,59]. As a soft phase, β grains promoted compatible deformation between the γ and α_2_ phase. Then, the material’s solid-state phase transformation roughly followed the path β→β + α→α_2_. The β phase transformed to the α phase when the alloy was cooled from the β phase field to room temperature. This transformation could occur in a diffusion-less martensitic mode or through a composition-invariant massive transformation involving short- range diffusion; alternatively, it could occur upon precipitating phase α from phase β. In addition to the β-to-α massive transformation, transformation of the α phase to the γ phase could also occur. The α phase undergoes an orderly transformation and finally transforms into α_2_ [58]. Finally, a microstructure containing deformed α_2_ grains and γ grains was obtained (Figure 9). 

**Figure 10:**
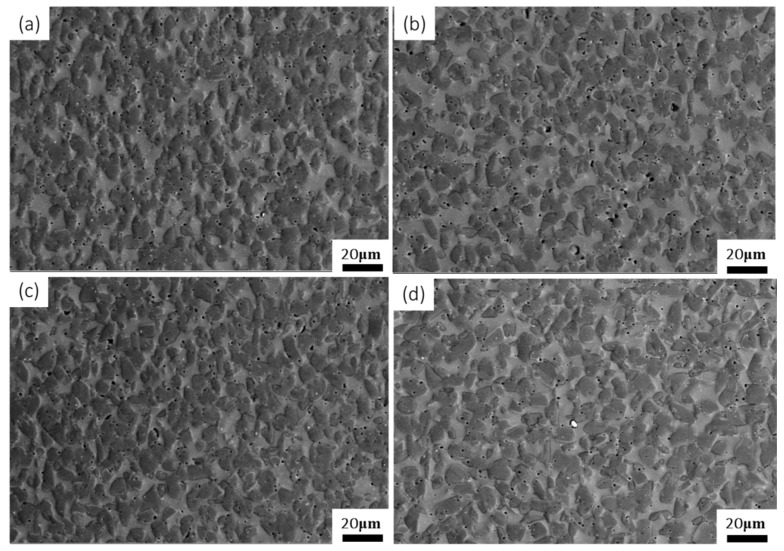
Microstructures of Ti43Al9V0.3Y specimen deformed in compression at 50% strain: (**a**) 1000 °C/0.001 s^−1^; (**b**) 1000 °C/0.01 s^−1^; (**c**) 1000 °C/0.1 s^−1^; (**d**) 1100 °C/0.1 s^−1^.

**Figure 11:**
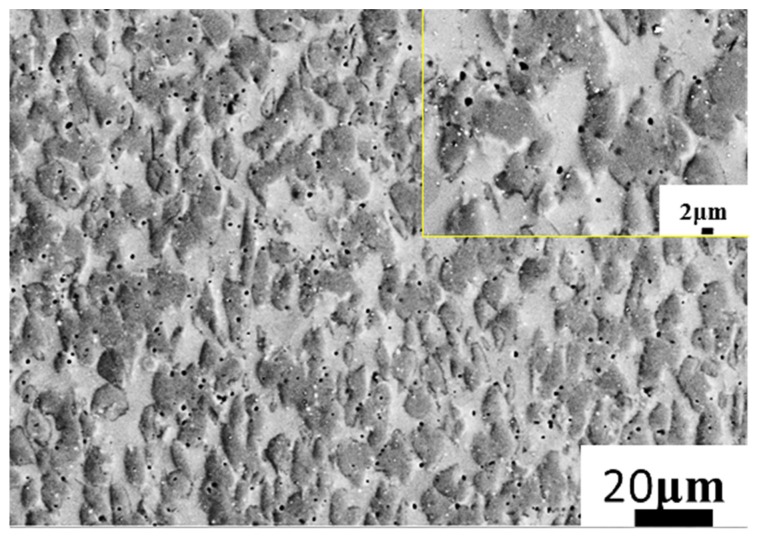
Microstructures of Ti43Al9V0.3Y specimen deformed in compression at 80% strain/1100 °C/0.1 s^−1^.

When the deformation temperature was below 1200 °C, the microstructure consisted of fine equiaxed γ (dark phase) and B2 (gray phase) grains (Figure 10 and Figure 11). Similar to the initial material (Figure 1a), the shape of the grains did not change significantly, and the size of the grains did not change. The dislocation density inside the grain was relatively low, indicating that DRX was the main deformation mechanism. The grain boundary slip may have also contributed to deformation, due to the inner part of the β grain being harder than the grain boundary [60,61]. No significant effect of strain rate on recrystallization was observed in this study, and the microstructure was still composed of fine equiaxed grains. γ increased slightly, but the grain size did not change much (Figure 10).

The IPP software was used to calculate the content of each phase. As the deformation rate increased, the percentage of the γ grain (dark phase) went from 73.9% ± 4.6% (Figure 10a) to 74.5% ± 4.1% (Figure 10b) to 89.6% ± 4.5% (Figure 10c). This implies that the percentage of γ grain increased. However, upon increasing temperature, the grain size increased (7 μm in Figure 10c and 11 μm in Figure 10d) and more B2 (gray phase) was obtained (10.4% B2 in Figure 10c and 22.7% B2 in Figure 10d).

Figure 10d and Figure 11 show the effect of strain on the microstructure. The microstructure still consisted of fine γ grain and B2 grain. In addition, the IPP software was used to calculate the content of each phase. The γ phase content decreased from 77.3% (Figure 10d) to 69.4% (Figure 11).

**Figure 12:**
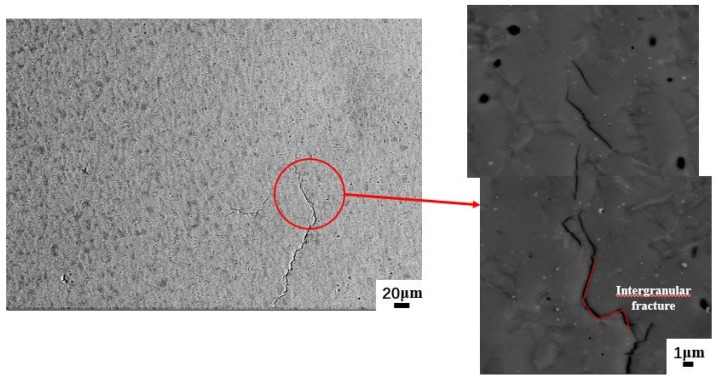
Microstructure of Ti43Al9V0.3Y specimen deformed in compression at 50% strain/1200 °C/0.1 s^−1.^

At 50% strain/1200 °C/0.1 s^−1^, obvious crack initiation and expansion could be observed (Figure 12). The cracks exhibited intergranular fracture characteristics.

**Figure 13:**
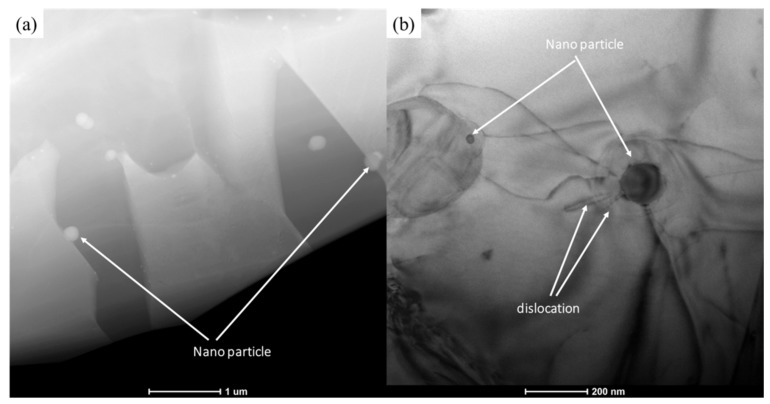
Microstructures of Ti43Al9V0.3Y specimen deformed in compression at 80% strain/1100 °C/0.1 s^−1^: (**a**) precipitates are mostly distributed on grain boundaries; (**b**) dislocations around the precipitate.

**Figure 14:**
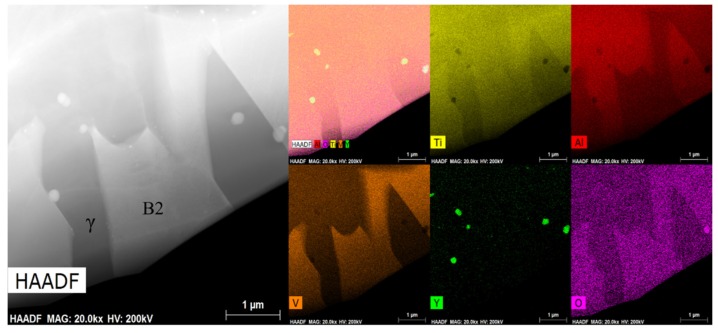
High angle annular dark field (HADDF) of Ti43Al9V0.3Y specimen deformed in compression at 80% strain/1100 °C/0.1 s^−1^.

In Figure 13, the microstructure consisted of equiaxed fine grains (Figure 13a). Dislocations surrounded the nanoparticles (Figure 13b). The dislocation networks in grains were not dense. This is because the material underwent dynamic recrystallization during deformation, and dynamic recrystallization eliminated a large number of dislocations, which was conducive to the subsequent deformation. DRV and DRX were the two main softening mechanisms in the high-temperature deformation [62]. DRX can be divided into three types [60]: geometric DRX (GDRX), continuous DRX (CDRX), and discontinuous DRX (DDRX). In Figure 13, bulging and serrated grain boundaries could be observed, which implied the appearance of DDRX [63]. At the same time, since the grain was very fine and uniform, the grain diameter was less than 10 μm, which enabled grain boundary slip in high-temperature deformation. In Figure 14, the white crystal grains contained more V, which were determined to be the B2 phase. The gray phase was the γ phase. Nanoparticles were mostly distributed in γ grains or on grain boundaries. This hindered the deformation of γ grains. Therefore, the deformation of the material may have been mainly contributed by the β phase. Nanoparticles mainly contained Y and O elements; thus, they might have been Y_2_O_3_. The nanoprecipitate was distributed on the matrix and it was surrounded by d dislocation lines. After encountering the grain boundary and other precipitates, the dislocation line was plugged in front of these obstructions (Figure 14). These precipitates increased the strength of the material by hindering the grain boundary motion and dislocation slip. However, these precipitates increased the deformation resistance when the material was deformed at high temperature, which was not conducive to material deformation.

## 4. Conclusions

The main conclusions from this work are as follows:

1. The stress exponent and activation energy of PM Ti43Al9V0.3Y alloys with fine equiaxed γ and B2 grain microstructure were 2.78 and 295.86 kJ/mol, respectively.
2. Reasonable hot working parameters at 80% strain were 1100–1200 °C/≤1 s^−1^. Furthermore, suitable hot working parameters at 50% strain were 1150–1200 °C/≤1 s^−1^ and 1000–1200 °C/≤0.05 s^−1^.
3. The microstructure evolution was found to be dependent on temperature, strain, and strain rate. When the deformation temperature was 1200 °C, the α phase mainly replaced the β phase, leading to a decrease in hot workability. Increases in temperature and strain both led to a decrease in γ.
4. The β phase is important for high-temperature deformation. Moreover, DRX contributes to the major deformation.

## Figures and Tables

**Table 1:** The actual composition of the initial material.

Elements	Ti	Al	V	Y
Content (at.%)	47.07	44.28	8.37	0.27

**Table 2:** Hot compressive parameters for test.

Items	Hot Compressive Parameters
Temperature (°C)	1000, 1100, 1200
Strain rate (s^−1^)	0.001, 0.010, 0.100, 1.000
Reduction in height (%)	50, 80
